# Current progress on pathogenicity‐related transcription factors in *Fusarium oxysporum*

**DOI:** 10.1111/mpp.13068

**Published:** 2021-05-09

**Authors:** Qussai Zuriegat, Yuru Zheng, Hong Liu, Zonghua Wang, Yingzi Yun

**Affiliations:** ^1^ State Key Laboratory of Ecological Pest Control for Fujian and Taiwan Crops College of Life Sciences Fujian Agriculture and Forestry University Fuzhou China; ^2^ Fujian Institute for Food and Drug Quality Control Fuzhou China; ^3^ College of Resources and Environment Fujian Agriculture and Forestry University Fuzhou China; ^4^ Institute of Oceanography Minjiang University Fuzhou China

**Keywords:** *Fusarium oxysporum*, pathogenicity, targets, transcription factor, virulence factors

## Abstract

*Fusarium oxysporum* is a well‐known soilborne plant pathogen that causes severe vascular wilt in economically important crops worldwide. During the infection process, *F*. *oxysporum* not only secretes various virulence factors, such as cell wall‐degrading enzymes (CWDEs), effectors, and mycotoxins, that potentially play important roles in fungal pathogenicity but it must also respond to extrinsic abiotic stresses from the environment and the host. Over 700 transcription factors (TFs) have been predicted in the genome of *F. oxysporum,* but only 26 TFs have been functionally characterized in various formae speciales of *F. oxysporum*. Among these TFs, a total of 23 belonging to 10 families are required for pathogenesis through various mechanisms and pathways, and the zinc finger TF family is the largest family among these 10 families, which consists of 15 TFs that have been functionally characterized in *F. oxysporum*. In this review, we report current research progress on the 26 functionally analysed TFs in *F. oxysporum* and sort them into four groups based on their roles in *F. oxysporum* pathogenicity. Furthermore, we summarize and compare the biofunctions, involved pathways, putative targets, and homologs of these TFs and analyse the relationships among them. This review provides a systematic analysis of the regulation of virulence‐related genes and facilitates further mechanistic analysis of TFs important in *F. oxysporum* virulence.

## Graphical abstract

The 26 characterized transcription factors with different signalling pathways and cellular processes are associated with virulence in *Fusarium oxysporum*.

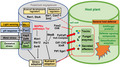

## 1 INTRODUCTION

*Fusarium oxysporum* is a filamentous plant‐pathogenic fungus that causes root rot, wilting, and necrosis in a large number of host plants and is ranked fifth out of the top 10 plant pathogens of scientific/economic importance (Dean et al., 2012; Geiser et al., 2013). This soilborne asexual fungus is known to include both pathogenic (plant, animal, and human) and nonpathogenic strains (Leslie & Summerell, 2006). Plant‐pathogenic strains have been divided into formae speciales based on host specificity (Lievens et al., 2008), some of which can be further divided into races or pathotypes based on their capacity to infect different cultivars (Table 1). *F. oxysporum* is classified into more than 100 formae speciales based on host species specificity, such as the tomato pathogen *F. oxysporum* f. sp. *lycopersici* (Fol), the banana pathogen *F. oxysporum* f. sp. *cubense* (Foc), the common bean pathogen *F. oxysporum* f. sp. *phaseoli* (Fop), and the melon pathogen *F. oxysporum* f. sp. *melonis* (Fom) (Takken & Rep, 2010). Currently, Royal Botanic Gardens (Kew) (https://www.kew.org) lists 124 formae speciales of *F. oxysporum*, whereas MycoBank (http://www.mycobank.org) lists 127.

**TABLE 1:** Examples of formae speciales in the plant pathogen *Fusarium oxysporum*

Formae specialis	Races	Host plant
*lycopersici*	I‐1, I‐2, and I‐3	Tomato
*cubense*	1, 2, subtropical race 4, and tropical race 4	Banana
*cucumerinum*	1, 2, and 3	Cucumber
*melonis*	0, 1, 2, 1.2Y, and 1.2W (previously 1 to 4)	Melon
*niveum*	0, 1, 2, and 3	Watermelon, melon
*melongenae*	None	Eggplant
*conglutinans*	1, 2 (syn. f. sp. *raphani*), 3 (syn. f. sp. *matthioli* race 1), 4 (syn. f. sp. *matthioli* race 2), and 5	Cabbage
*cepae*	None	Onion, green onion
*apii*	1, 2, 3, and 4	Celery

Following penetration of *F. oxysporum* through plant roots, its colonization in the vascular system disrupts water‐conducting xylem vessels, leading to wilting symptoms and death of the plant (Yadeta & Thomma, 2013). During the infection process, *F. oxysporum* employs a number of secretion systems and elicits a variety of virulence factors, such as mycotoxins, effector proteins, and plant cell wall‐degrading enzymes (CWDEs), to subvert target host cells (Ma et al., 2013). CWDEs, such as polygalacturonases, pectate lyases, xylanases, and cutinases, may contribute to pathogenesis by degrading waxes, cuticles, and cell walls to induce tissue invasion and pathogen dispersal (Ma et al., 2013). Additionally, *F. oxysporum* can produce mycotoxins, including beauvericin (Li et al., 2013), fusaric acid (López‐Díaz et al., 2018), and fumonisins (Rheeder et al., 2002), which contribute to pathogenicity in hosts. Beauvericin and fusaric acid have been associated with Foc, causing banana plantlets to wilt, decay, and die in vitro (Li et al., 2013). In Foc tropical race 4 (TR4), fusaric acid plays a key role as a positive virulence factor, particularly at the early stage of disease development (Liu et al., 2020). Beauvericin is a mycotoxin produced by many *Fusarium* species and by *Beauveria bassiana* that induces DNA fragmentation and apoptosis by disrupting mitochondrial pathways (Mallebrera et al., 2018). In Fol, beauvericin reduces the level of ascorbic acid in tomato cells, leading to collapse of the ascorbate system and protoplast death (Paciolla et al., 2004).

In addition to CWDEs and mycotoxins, *F. oxysporum* produces effectors known as Six (secreted in xylem) proteins, a group of cysteine‐rich effectors identified from the xylem sap of tomato after inoculation with pathogens. To date, 14 Six effectors have been identified. In Fol, some *SIX* genes have been found to play significant roles in determining host specificity. For instance, *SIX4* (*AVR1*), *SIX3* (*AVR2*), and *SIX1* (*AVR3*) act as avirulence genes that interact with the tomato resistance genes *I‐1* (Immunity‐1), *I‐2*, and *I‐3*, respectively (Taken & Rep, 2010). *SIX* genes have been identified in a range of *F. oxysporum* formae speciales, but each forma specialis of the fungus has a specific combination of these *SIX* genes that can be used to predict *F. oxysporum* host specificity (van Dam et al., 2016). In Fol, the *SIX* genes are located on lineage‐specific chromosomes known as accessory chromosomes that are enriched in transposable elements (TEs) and play a role in host specialization (Ma et al., 2010). Moreover, accessory chromosomes can be horizontally transferred to another strain, which is associated with the host‐specific pathogenicity of *F. oxysporum* (Ma et al., 2010; van Dam et al., 2017).

Transcription factors (TFs) are the main players in transcriptional regulation of pathways implicated in *F. oxysporum* virulence. During infection, *F. oxysporum* connects sensing of host cues to transcriptional reprogramming by activating a number of TFs that govern the physiological adaptation, pathogenesis, and growth of the fungus to adapt to the host environment (Guo et al., 2014). Previously, the genome of Fol was sequenced, and approximately 5% (genome size) of the genes were found to encode TFs, making them the largest protein family in *F. oxysporum* (Ma et al., 2010). Genomic analysis of both Foc race 1 and Foc race 4 showed that the genome structures are highly syntenic with that of Fol and that Foc race 1 encodes 729 TFs compared to 793 for Foc race 4 (Guo et al., 2014). To date, only 26 TFs have been functionally analysed in *F. oxysporum,* but comparison and summarization of the biological functions and mechanisms of these TFs are limited.

## 2 MAJOR FAMILIES OF TFs IN *F*. *OXYSPORUM*

TFs are grouped into well‐defined structural families on the basis of their DNA‐binding domains (DBDs). In eukaryotes, including filamentous fungi, the largest of these are the zinc finger (ZF), homeodomain (HD), basic leucine zipper (bZIP), and basic helix‐loop‐helix (bHLH) families (Meshi & Iwabuchi, 1995; Pabo & Sauer, 1992). In the Fungal Transcription Factor Database (FTFD; http://ftfd.snu.ac.kr), 795 putative TFs classified into 43 families were found in Fol (Park et al., 2008). The majority of Fol TFs belong to the Zn(II)2Cys6 fungal binuclear cluster family (370); other types of TFs include C2H2 zinc finger (73), homeodomain‐like (63), bZIP family (55), bHLH TF family (46), nucleic acid‐binding, OB‐fold (42), HMG (39), winged helix repressor DNA‐binding (35), and Myb (22) TFs, among others (Park et al., 2008). Among them, only 26 TFs belonging to 10 families have been functionally analysed (Table 2).

**TABLE 2:** Summary of the characterized *Fusarium oxysporum* transcription factors (TFs) discussed in this review

Family	TF	Domains^a^	*Fusarium oxysporum* species	Virulence in planta	Reference
Zn(II)2Cys6	Ctf1	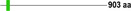	*F. oxysporum* f. sp. *lycopersici* (Fol)	Reduced	Rocha et al. (2008)
	Ctf2	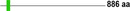	Fol	Reduced	Rocha et al. (2008)
	Fow2	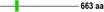	*F. oxysporum* f. sp. *melonis* (Fom)	Reduced	Imazaki et al. (2007)
			Fol	Reduced	
	Ftf1	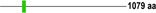	*F. oxysporum* f. sp. *phaseoli* (Fop)	Reduced	Ramos et al. (2007)
					van der Does et al. (2016)
			Fol		Zhao et al. (2020)
	XlnR	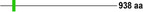	Fol	Fully virulent	Calero‐Nieto et al. (2007)
	Ebr1	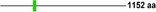	Fol	Reduced	Jonkers et al. (2014)
C2H2	FoCzf1	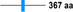	Fol	Reduced	Yun et al. (2019)
	Con7‐1	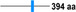	Fol	Reduced	Ruiz‐Roldán et al. (2015)
	PacC	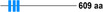	Fol	More virulent	Caracuel et al. (2003)
	ZafA	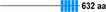	Fol	Reduced	López‐Berges 2019
	St12	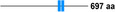	Fol	Reduced	Rispail and Di Pietro (2009)
			Fop		Asunción García‐Sánchez et al. (2010)
bZIP	HapX	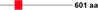	Fol	Reduced	López‐Berges et al. (2012)
	FoAtf1	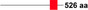	Fol	Reduced	Qi et al. (2013)
	MeaB	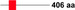	Fol	Reduced	López‐Berges et al. (2010)
PHD	Cti6	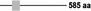	Fol	Reduced	Michielse, van Wijk, Reijnen, Cornelissen, et al. (2009)
	Snt2	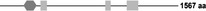	Fom	Reduced	Denisov et al. (2011)
GATA	Wc1	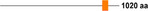	Fol	Reduced	Ruiz‐Roldán et al. (2008)
	Fnr1	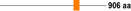	Fol	Reduced	Divon et al. (2006)
Velvet	VeA	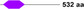	Fol	Reduced	López‐Berges et al. (2013)
	VelB	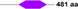	Fol	Reduced	López‐Berges et al. (2013)
MADS‐bo	Rlm1	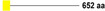	*F*. *oxysporum* f. sp. *cubense* (Foc)	Reduced	Ding et al. (2020)
Gti1/Pac2	Sge1	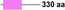	Fol	Avirulent	Michielse, van Wijk, Reijnen, Manders et al. (2009)
			Foc tropical race 4		Hou et al. (2018); Zhao et al. (2020)
			Foc race 1		Gurdaswani et al. (2020)
APSES	StuA	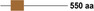	Fom	Fully virulent	Ohara and Tsuge (2004)
	Swi6	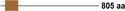	Fol	Reduced	Ding et al. (2018)
HSF	FoSkn7	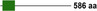	Foc race 4	Reduced	Qi et al. (2019)
‐	Ren1	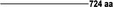	Fom	Fully virulent	Ohara et al. (2004)

^a^ The light green rectangle represents the Zn(II)2Cys6 domain; the light blue rectangle represents the C2H2 domain; the red rectangle represents the bZIP domain; the grey rectangle or hexagon represents the PHD and BAH domains, respectively; the orange rectangle represents the GATA domain; the purple hexagon represents the Velvet family domain; the yellow rectangle represents the MADS‐box domain; the pink rectangle represents the Gti1/Pac2 family domain; the brown rectangle represents the APSES family domain; the dark green rectangle represents the HSF domain. aa, amino acids.

The zinc finger (ZF) motif is the most abundant type of TF, is found in all living organisms, and forms one of the largest families of TFs in eukaryotes (Seetharam & Stuart, 2013). These proteins contain two‐stranded antiparallel β‐sheets and one α‐helix, and based on their conserved cysteine (Cys) and histidine (His) residues that fold into a finger‐like structure, they are classified into nine subfamilies: Cys2/His2‐type (C2H2), C3H, C3HC4, C2HC5, C4HC3, C2HC, C4, C6, and C8 (Cassandri et al., 2017). Among these subfamilies, the C6 type of zinc cluster protein is found exclusively in yeast and in filamentous fungi, and contains a well‐conserved CysX2CysX6CysX5‐12CysX2CysX6‐8Cys motif with cysteines that bind to two zinc atoms; TFs of this type are referred to as binuclear ZF Zn(II)2Cys6 proteins (Klimova et al., 2014). In *F. oxysporum*, a total of six Zn(II)2Cys6 TFs have been functionally analysed, namely, Ctf1, Ctf2, Fow2, Ftf1, XlnR, and Ebr1 (Table 2) (Bravo‐Ruiz et al., 2013; Calero‐Nieto et al., 2007; Imazaki et al., 2007; Jonkers et al., 2014; Ramos et al., 2007). In the C2H2 zinc finger domain, the presence of a zinc atom with two conserved cysteine residues at one end of the β‐sheet and two conserved histidine residues at the α‐helix C‐terminus ensure the stability of the zinc finger structure (Wolfe et al., 2000). In *F. oxysporum*, a total of five C2H2 ZFs have been identified and functionally tested, namely, PacC, ZafA, Ste12, FolCzf1, and Con7‐1 (Table 2) (Asuncion Garcia‐Sanchez et al., 2010; Caracuel et al., 2003; López‐Berges, 2020; Ruiz‐Roldan et al., 2015; Yun et al., 2019). GATA proteins, a family of GATA‐binding zinc finger transcription factors, play diverse functions in fungi and consist of one or two zinc‐binding modules with four cysteines embedded in the sequence Cys‐X2‐Cys‐X17/18‐Cys‐X2‐Cys and an adjacent basic region (Teakle & Gilmartin, 1998). In *F. oxysporum*, a total of two GATA transcription factors, Fnr1 and Wc1, have been functionally analysed (Divon et al., 2006; Ruiz‐Roldan et al., 2008). The plant homeodomain (PHD) zinc finger domain has a C4HC3‐type motif, initially described in 1993 as a functional domain present in the *Arabidopsis* homeodomain protein HAT3.1 (Schindler et al., 1993). In *F. oxysporum*, a total of two PHD‐containing transcription factors, Snt2 and Cti6, have been functionally tested (Denisov et al., 2011; Michielse, van Wijk, Reijnen, Cornelissen, et al., 2009).

Basic leucine zipper (bZIP) transcription factors are highly conserved in eukaryotes and form one of the largest families of dimerizing TFs (Ellenberger, 1994). bZIP family proteins are characterized as having a basic region for DNA binding and a leucine zipper region for protein dimerization consisting of up to nine heptad repeats with a leucine every seven amino acids, thereby allowing two monomers to “zip up” when forming the protein dimer (Ellenberger, 1994; Ellenberger et al., 1992). In *F. oxysporum*, a total of three bZIP TFs have been functionally analysed: FoAtf1, MeaB, and HapX (López‐Berges et al., 2010, 2012; Qi et al., 2013).

Velvet complex proteins are highly conserved in filamentous fungi and include VeA (VelA), VelB, VelC, and LaeA proteins (Bayram et al., 2008). These proteins have a 150‐amino acid region known as the velvet domain, which is thought to be a key regulator of diverse cellular processes, such as secondary metabolism and asexual or sexual sporulation (Bayram & Braus, 2012). In *F. oxysporum,* a total of two transcription factors belonging to this family have been functionally analysed, VeA and VelB (López‐Berges et al., 2013).

The APSES protein family belongs to the basic helix‐loop‐helix (bHLH) TF class and is widely conserved in fungi. The first APSES members characterized were Asm1 in *Neurospora crassa*, Phd1 in *Saccharomyces cerevisiae*, Sok2 in *S. cerevisiae*, Efg1 in *Candida albicans*, and StuA in *Aspergillus nidulans*, and these proteins are involved in various biological processes, such as sporulation, cellular differentiation, mycelial growth, secondary metabolism, and virulence in fungi (Zhao et al., 2015). In *F. oxysporum,* two transcription factors belonging to this family, StuA and Swi6, have been functionally analysed (Ding et al., 2018; Ohara & Tsuge, 2004).

Only one transcription factor each from the MADS‐box, Pac2/Gti1, and HSF (heat shock factor) families has been analysed in *F. oxysporum*. MADS is an acronym of the initials of MINICHROMOSOME MAINTENANCE 1 (MCM1) from *S. cerevisiae*, AGAMOUS (AG) from *Arabidopsis*, DEFICIENS (DEF) from *Antirrhinum majus*, and S for serum response factor (SRF) from *Homo sapiens* (Yanofsky et al., 1990). In *F. oxysporum*, MADS‐box Rlm1 is the only TF that has been functionally tested (Ding et al., 2020). Gti1/Pac2 is a conserved fungal protein family that regulates morphogenic transition and controls toxin production and pathogenesis in fungi (Chen et al., 2014). Pac2 and Gti1 were the first members characterized in this family of TFs. Sge1 is the only member of this family that has been functionally analysed in *F. oxysporum* (Michielse, van Wijk, Reijnen, Manders, et al., 2009). Heat shock factor‐type transcriptional regulators bind specifically to heat shock sequence elements (HSEs) throughout the genome (Guertin & Lis, 2010). Their activators have a role in maintaining cell wall integrity and regulating the osmotic/oxidative stress response (OSR) in *S. cerevisiae*, where it is part of a two‐component signal transduction system. FoSkn7 is the only member of this family that has been functionally analysed in *F. oxysporum* (Qi et al., 2019).

## 3 TRANSCRIPTIONAL REGULATION OF DIRECT VIRULENCE FACTORS IN *F. OXYSPORUM*, INCLUDING EFFECTORS, CWDEs, AND MYCOTOXINS

Effectors, CWDEs, and mycotoxins are assumed to be important direct virulence factors that are transcriptionally regulated during colonization of *F. oxysporum* in host tissues (van Dam et al., 2017; Liu et al., 2020; Ma et al., 2010). Among the 26 functionally analysed TFs in *F. oxysporum*, Sge1 and Ftf1 regulate the expression of effector genes. Xln1, Ctf1, and Ctf2 are responsible for regulation of CWDE activities, while FolCzf1 and the velvet complex act as regulators of mycotoxin production. Research progress on the above TFs will be introduced below.

### 3.1 Sge1 and Ftf1 regulate effector‐encoding genes in *F. oxysporum*

Sge1 (Six Gene Expression 1), the ortholog of Wor1, was first identified in Fol (Michielse, van Wijk, Reijnen, Manders, et al., 2009). In Fol, the *SGE1* deletion mutant (ΔSGE1) almost loses virulence against tomato plants, and deletion of *SGE1* affects parasitic growth within xylem tissue but not colonization of the root surface or root penetration (Michielse, van Wijk, Reijnen, Manders, et al., 2009). Sge1 is thought to be implicated in regulating the expression of the effector genes *SIX1*, *SIX2*, *SIX3*, and *SIX5* in Fol (Michielse, van Wijk, Reijnen, Manders, et al., 2009). Homologs of *SGE1* in Foc tropical race 4 (Foc TR4) and race 1 are also required for regulation of virulence in banana roots. An in planta RNAi (siRNA) approach using host‐induced silencing of the *SGE1* gene of Foc TR4 in banana plants confirmed that the *SGE1* gene is directly involved in the virulence of Foc (Fernandes et al., 2016). In other *Fusarium* species, such as *F. graminearum* and *F. verticillioides*, Sge1 is thought to regulate the expression of effectors and secondary metabolite biosynthetic genes (Brown et al., 2014; Jonkers et al., 2012). Homologs of Sge1 in other fungal plant pathogens, including *Botrytis cinerea* (Reg1), *Verticillium dahliae* (VdSge1), *Ustilago maydis* (Ros1), and *Magnaporthe oryzae* (Gti1), are also required for virulence (Michielse et al., 2011; Santhanam & Thomma, 2013; Tollot et al., 2016). In *U. maydis,* using ChIP‐seq and RNA‐seq, Tollot et al. identified 128 putative effector genes among the targets of Ros1. The above results suggest a conserved function of Sge1 homologs in regulation of pathogenicity and effectors among fungal plant pathogens.

Fusarium transcription factor (*FTF*) is one of the expanded gene families in the accessory chromosomes of *F. oxysporum*. The *FTF* gene family, belonging to Zn2Cys6 family TFs, comprises a single‐copy gene *FTF2*, which is present in all filamentous ascomycetes. Several copies of *FTF1* are exclusively present in *F. oxysporum* (Nino‐Sanchez et al., 2016). The TFs *FTF1* and *FTF2* are involved in regulating the expression of *SIX1* and one of its regulators, *SGE1* (Michielse, van Wijk, Reijnen, Manders, et al., 2009). In Fol, *FTF1* paralogs are located on accessory chromosomes and *FTF2* (*FTF1* homolog) is present on a core chromosome. Deletion of accessory chromosome *FTF1* paralogs decreased the virulence of Fol toward the host (de Vega‐Bartol et al., 2011; Nino‐Sanchez et al., 2016; Schmidt et al., 2013), while deletion of *FTF2* did not affect the growth or sporulation of Fol but slightly reduced virulence toward the host. Similar to *SGE1*, deletion of *FTF1* in Fop does not affect the host penetration process but does impact successful colonization of Fop in the plant xylem, and Ftf1 positively regulates the effector genes *SIX1* and *SIX6*, both of which are present in the genome of highly virulent Fop strains but absent in the genome of weakly virulent strains (Michielse, van Wijk, Reijnen, Manders, et al., 2009; Nino‐Sanchez et al., 2016). A previous study based on expression profile analysis of Fol revealed that the transcription levels of both *SGE1* and *FTF1* increase during infection processes, and intrinsic expression of *FTF1* or *SGE1* induces expression of a large overlapping set of known *SIX* genes, including *SIX6*, *SIX9*, and *SIX13,* in Fol, suggesting interaction of these TFs (van der Does et al., 2016).

### 3.2 XlnR, Ctf1, and Ctf2 regulate CWDE‐encoding genes in *F. oxysporum*

In Fol, deletion of the *XLNR* gene resulted in a lack of transcriptional activation of xylanase genes both in culture and during infection of tomato plants, along with a reduction in extracellular xylanase activity. However, the *XLNR* mutants were still fully pathogenic on tomato plants (Calero‐Nieto et al., 2007). These results revealed that XlnR, the key transcriptional activator of xylanase genes, is not an essential virulence determinant in *F. oxysporum* (Calero‐Nieto et al., 2007). XlnR was originally reported as a xylanase gene regulator in *Aspergillus* spp. (van Peij et al., 1998). However, it has been revealed that XlnR regulates different gene sets in different fungal species. In *M. oryzae,* XlnR was found to regulate the expression of genes involved in the pentose catabolic pathway but not genes encoding hemicellulolytic enzymes (Battaglia et al., 2013). In *F. graminearum*, Xyr1, the homolog of XlnR, regulates xylanase but not cellulase secretion (Brunner et al., 2007).

Ctf1 and Ctf2 are two homologs involved in regulation of the lipolytic system in Fol (Bravo Ruiz et al., 2013). Single‐gene deletion mutants of *CTF1* or *CTF2* or double‐gene deletion of *CTF1* and *CTF2* in Fol led to reduced total lipase activity, and both Ctf1 and Ctf1 were found to positively regulate seven lipase genes, *LIP1*, *LIP2*, *LIP3*, *LIP10*, *LIP13*, *LIP 15*, and *LIP 22*. Ctf1 also showed negative regulation of the *LIP 1.2*, *LIP20*, and *LIP21* genes, while Ctf2 negatively affected the expression of *LIP1.2*. Furthermore, the above mutants all show reduced virulence in tomato, and these results indicated that the lipolytic system of Fol is important in pathogenicity (Bravo‐Ruiz et al., 2013). Homologs of Ctf1 and Ctf2 have been found in several filamentous fungi, including *A. nidulans*, *Nectria haematococca*, and *M. oryzae*, and these homologs have conserved functions in the lipolytic system (Hynes et al., 2006; Li et al., 2002; bin Yusof et al., 2014).

### 3.3 FolCzf1 and velvet complex regulate mycotoxin production in *F. oxysporum*

The C2H2 TF FolCzf1 plays an important role in early infection in *F. oxysporum* (Yun et al., 2019). Deletion of *FolCZF1* resulted in a loss of virulence and decreased fusaric acid production (Yun et al., 2019). A transcriptional profiling study further showed that FolCzf1 is involved in regulating fusaric acid biosynthesis genes (Yun et al., 2019). The homologs of FolCzf1, *F. graminearum* GzC2H003, and *M. oryzae GCF3* are highly expressed in the early infection stage and are required for the pathogenicity of *F. graminearum* and *M. oryzae* (Cao et al., 2016; Son et al., 2011). Furthermore, deletion of GzC2H003 does not affect the biosynthesis of the mycotoxin deoxynivalenol but leads to increased production of zearalenone (Son et al., 2011).

The heterotrimeric velvet complex is a conserved regulator of fungal development and secondary metabolism previously examined in other *Fusarium* pathogenic fungi, such as *F. graminearum*, *F. verticillioides*, and *F. fujikuroi* (Bayram et al., 2008; Jiang et al., 2012; Wiemann et al., 2010). In Fol, loss of the velvet components *VEA*, *VELB*, or *VELC* caused increased conidiation as well as changes in the shape and size of microconidia. VeA and LaeA are required for full virulence of Fol on tomato plants (Lopez‐Berges et al., 2013). In addition, the velvet protein complex is involved in expression of the gene cluster for beauvericin (Lopez‐Berges et al., 2013). However, in *F. graminearum*, disruption of the *FgVEA* gene causes an increase in conidial production, whereas conidial germination is delayed and a reduction in aerial hyphal formation, hydrophobicity, virulence, and deoxynivalenol production is observed (Jiang et al., 2011).

## 4 TRANSCRIPTIONAL REGULATION OF PATHOGENICITY UNDER ABIOTIC STRESS *IN F. OXYSPORUM*

Phytopathogenic fungi establish parasitic or growth‐promoting relationships depending on the availability of micronutrients and surrounding abiotic stresses. Essential nutrients include microelements, such as iron and zinc, that are needed in small amounts, while other micronutrients, such as nitrogen, must be obtained in large amounts from the surroundings (Abbaspour et al., 2014; Divon et al., 2006; Lopez‐Berges et al., 2010). Nitrogen availability can be a limiting factor for growth and basic metabolic processes in *F. oxysporum* (Divon et al., 2006) and plays a key role in signal transduction to activate the expression of virulence genes in *F. oxysporum* (Lopez‐Berges et al., 2010; Tudzynski, 2014). Previous studies have confirmed a link between nitrogen limitation stress and virulence in plant pathogens. For instance, in the presence of a preferred nitrogen source, suppression of virulence functions occurs during hyphal fusion and root adhesion in Fol (Lopez‐Berges et al., 2010). Similarly, preferred nitrogen sources were also able to block Fol penetration of cellophane, an in vitro method used to mimic host penetration (Lopez‐Berges et al., 2010). Two TFs have been reported as nitrogen regulators in *F. oxysporum*, a GATA‐type nitrogen regulator named Fnr1 and a bZIP TF named MeaB.

Biological responses to abiotic stresses, including heavy metals, pH changes, light, and oxidative pressure, also affect the pathogenicity of *F. oxysporum*. During the infection process, *F. oxysporum* must adapt to a limited availability of metals. Metal homeostasis requires elaborate mechanisms to maintain the balance between uptake, storage, and consumption (Wallace, 2016). The TFs Hapx and ZafA are required for regulation of iron and zinc homeostasis in *F. oxysporum*, respectively. Plant infections caused by fungi are often associated with an increase in the pH of the surrounding host tissue. Extracellular alkalization is thought to contribute to the fungal pathogenesis of *F. oxysporum* (Masachis et al., 2016). PacC is an important pH‐responsive TF in *F. oxysporum*. Another factor affecting the virulence of *Fusarium* pathogens is light. Light is a signal from the environment that regulates many fungal development processes. Photoreceptors sense light and generate signals that stimulate cellular responses, such as carotenoid biosynthesis, spore formation, and phototropism (Yu & Fischer, 2019). Recent studies have demonstrated that the presence of a photosensor component is needed not only for ecological adaptation of the pathogen but also for regulation of virulence factor expression (Tang et al., 2020). In *F. oxysporum*, the TF WC‐1 is the key element involved in light signal transduction. The oxidative burst, a defence strategy observed in most host plants during fungal invasion, consists of rapid production of large amounts of reactive oxygen species to control fungal penetration during root colonization (Zeilinger et al., 2016). Therefore, *F. oxysporum* has evolved different mechanisms to detoxify reactive oxygen species to minimize damage during invasion of the host. FoAtf1 and FoSkn7 are regulators of oxidative stress response genes in *F. oxysporum*.

Several conserved signal transduction pathways have been found to be involved in regulation of the environmental response in fungi, and mitogen‐activated protein kinase (MAPK) cascades are one of the well‐studied pathways. MAPK cascades are characterized by a three‐tiered module comprising a MAP kinase kinase (MAPKKK), a MAP kinase (MAPKK), and a MAPK that is activated by dual phosphorylation of conserved threonine and tyrosine residues within the activation loop (Jiang et al., 2018). Sequential activation of the MAPK cascade eventually results in activation of TFs and the expression of specific sets of genes in response to environmental stimuli (Zhao et al., 2007). Three MAPK pathways (Fmk1, Mpk1, and Hog1, orthologs to Fus3/Kss1, Mpk1, and Hog1 in yeast, respectively), have been identified in ascomycetes, including *F. oxysporum* (Jiang et al., 2018; Segorbe et al., 2017). In *F. oxysporum*, both Fmk1 and Mpk1 regulate responses to cell wall and heat stresses, and Hog1 probably negatively controls the activation of Fmk1 and Mpk1 (Segorbi et al., 2017). Three downstream MAPK pathway TFs (Rlm1, Swi6, and Ste12) of MAPK pathways have been demonstrated to contribute to the stress response and found to be involved in the pathogenesis of *F. oxysporum*.

### 4.1 Fnr1 and MeaB are involved in the nitrogen catabolic process and the pathogenicity of *F. oxysporum*

GATA‐ZF TF AreA homologs have been identified as regulators of nitrogen nutrition genes in filamentous fungi, and the homolog of AreA in Fol was named Fnr1 (Fusarium nitrogen regulator 1) (Divon et al., 2006). Fnr1 disruption mutants showed a reduced ability to utilize several secondary nitrogen sources, whereas growth on favourable nitrogen sources was not affected (Divon et al., 2006). Deletion of *FNR1* led to an obvious reduction in the pathogenicity of Fol on tomato and abolished the in vitro expression of nutrition genes during the early phase of infection, suggesting that Fnr1 mediates adaptation to nitrogen‐poor conditions in planta through regulation of secondary nitrogen acquisition and acts as a determinant for fungal fitness during infection (Divon et al., 2006). In *S. cerevisiae*, when TOR (target of rapamycin) kinase is inactive under starvation conditions, GATA‐ZF TF Gln3 can be dephosphorylated and imported into the nucleus to activate the transcription of genes that are required for adaptation of *S. cerevisiae* to less preferred nitrogen sources. In *F. graminearum*, FgAreA could fully complement the budding yeast *GLN3* deletion mutant, and *FgAREA* disruption showed phenotypes similar to those of the TOR pathway component *FgPPG1* mutant, suggesting that FgAreA is one of the downstream components in the TOR signalling pathway. However, the relationship between Fnr1 and TOR kinase has rarely been investigated in *F. oxysporum*.

Nitrogen utilization is a highly regulated process; if the preferred nitrogen source (such as ammonium) is available, *A. nidulans* TF MeaB induces transcriptional activation of the corepressor of AreA, NmrA, to inhibit the activation of AreA (Tudzynski, 2014). In Fol, loss of MeaB led to reduced growth on secondary nitrogen sources and very poor growth on ammonium as the sole nitrogen source, and these phenotypes were similar to those reported for the *MEAB* mutant of *A. nidulans*. Introduction of *A. nidulans MEAB* into the Fol *MEAB* mutant restored wild‐type growth on all nitrogen sources tested, suggesting that homologs of MeaB in nitrogen utilization are highly conserved between *F. oxysporum* and *A. nidulans*. In addition, tomato supplied with ammonium nitrate showed a significant delay in symptom development, but in plants infected with the *MEAB* mutant the nitrogen source had no significant effect on the severity of disease symptoms, suggesting that ammonium negatively regulates plant infection by *F. oxysporum* in a MeaB‐dependent manner.

### 4.2 HapX and ZafA regulate *F. oxysporum* metal homeostasis and pathogenicity

In Fol, *HAPX* plays an important role in iron homeostasis (López‐Berges et al., 2012). Deletion of *HAPX* activates the genes involved in iron‐consuming pathways but does not affect iron uptake, which leads to defective growth under iron starvation conditions (López‐Berges et al., 2012). However, deletion of HapX homologs does not alter the transcription of iron acquisition genes in Fol. Furthermore, HapX contributes to iron competition of Fol against siderophore‐producing pseudomonads in the tomato rhizosphere (López‐Berges et al., 2012). The *HAPX* deletion mutant also shows decreased pathogenicity toward tomato plants (Lopez‐Berges et al., 2012). In previous studies, homologs of HapX were also thought to be involved in the virulence of several human fungal pathogens, such as *A. fumigatus* and *C. albicans* (Chen et al., 2011; Schrettl et al., 2010). HapX homologs have been shown to be crucial for maintaining iron homeostasis in several filamentous ascomycetes, including *A. nidulans* (Hortschansky et al., 2007), *F. graminearum* (Wang et al., 2019), and *V. dahliae* (Wang et al., 2018), and in the yeast ascomycete *C. albicans* (Chen et al., 2011) and the yeast basidiomycete *Cryptococcus neoformans* (Jung et al., 2010), suggesting functional conservation of *HAPX* in fungi.

In Fol*, ZAFA* expression is induced under zinc‐limiting conditions and repressed by zinc at early stages of plant infection, which regulates the expression of many genes in response to zinc deficiency, including high‐affinity membrane zinc transporters, such as Zrt1, Zrt2, Zrt3, Fet4, and Zrc1 (López‐Berges, 2020). ZafA is also required for full virulence of Fol in plant and animal hosts (López‐Berges, 2020). In the opportunistic fungal pathogen *A. fumigatus*, the homolog of ZafA, AoZafA, also plays an essential role in maintaining zinc homeostasis, and AoZafA was found to be regulated by pH and influenced by the PacC transcriptional regulator (Amich et al., 2009, 2010).

### 4.3 PacC regulates the pH response but is a negative regulator of *F. oxysporum* pathogenicity

The expression level of *PACC* is elevated in Fol grown under alkaline conditions and is almost undetectable under extreme acidic growth conditions. Gene deletion of *PACC* led to an acidity‐mimicking phenotype, resulting in poor growth at alkaline pH and increased acid protease activity, while overexpression of *PACC* led to the opposite phenotype. However, loss of *PACC* resulted in increased virulence in tomato roots, suggesting that Fol PacC acts as a negative regulator of virulence in plants, possibly by preventing transcription of acid‐expressed genes important for infection (Caracuel et al., 2003). The homologs of PacC in other filamentous fungi, including *A. nidulans*, *M. oryzae*, and *Fusarium* species, showed a conserved role in regulation of pH‐response genes (Bignell et al., 2005; Landraud et al., 2013). In addition, in *F. graminearum* and *F. verticillioides*, PacC homologs are also involved in secondary metabolites by regulating the production of trichothecene and fumonisins (Flaherty et al., 2003; Merhej et al., 2011).

### 4.4 Wc‐1 is involved in light signal transduction in *F. oxysporum*

White collar 1 (WC‐1) and WC‐2 are two key elements involved in light signal transduction that have been previously studied in *N. crassa* (Ballario et al., 1996; Liu et al., 2003). *WC1* gene deletion Fol mutants show aerial hyphal impairments, surface hydrophobicity, light‐induced carotenogenesis, photoreactivation after UV treatment, and up‐regulation of photolyase gene transcription, confirming the role of Wc‐1 in light response signalling (Ruiz‐Roldan et al., 2008). In addition, Wc1 is nonessential for the pathogenicity of Fol in plants but is required for full virulence in mammals (Ruiz‐Roldan et al., 2008). In addition, transcriptional activation of photolyase genes mediated by white collar complexes has previously been described in fungi such as *F. graminearum* and *C. neoformans* (Kim et al., 2015; Zhu & Idnum, 2018).

### 4.5 FoAtf1 and FoSkn7 regulate the oxidative stress response and *F. oxysporum* pathogenicity

The bZIP TF FoAtf1 has been characterized in Foc race 4. The *FoATF1* deletion mutant was highly sensitive to hydrogen peroxide compared with the wild‐type strain (Qi et al., 2013). In addition, FoAtf1 is thought to be involved in the reduction of extracellular enzyme activity and the transcription level of catalase. Furthermore, FoAtf1 has been shown to be involved in virulence by regulating the oxidative stress responses of Cavendish banana (*Musa* spp.) (Qi et al., 2013). In *M. oryzae*, MoAtf1 is involved in the oxidative stress response by regulating host‐derived reactive oxygen species levels and facilitating successful pathogen invasion of host tissues (Guo et al., 2010).

In Foc race 4, FoSkn7 is thought to be involved in growth and conidiation as well as in the reduction of Foc race 4 virulence (Qi et al., 2019). The *FoSKN7* deletion mutant is highly sensitive to oxidative stress (Qi et al., 2019). Previous work in *A. flavus* has revealed that Skn7 induces the expression of several antioxidant genes that provide resistance to hydrogen peroxide, including genes encoding peroxidase, catalase, and thioredoxin (Zhang et al., 2016; Vargas‐Perez et al., 2007). The functions of FoSkn7 are similar to those of Skn7 orthologs in *Alternaria alternata* and in other fungi that participate in the oxidative stress response, such as *Penicillium marnefei*, *C*. *glabrata*, and *S. cerevisiae* (Cao et al., 2009; Chen et al., 2012; Mulford & Fassler, 2011; Saijo et al., 2010).

### 4.6 Rlm1, Swi6, and Ste12 act as downstream TFs involved in MAPK pathways in *F. oxysporum*

Mpk1 is the key kinase in the conserved cell wall integrity (CWI) MAPK pathway, repairing the cell wall and maintaining cellular integrity, which is mediated by Swi4‐Swi6 cell cycle box‐binding factor (SBF) and the MADS‐box transcription factor Rlm1 in the budding yeast *S. cerevisiae* (Levin, 2011). In Foc, the homologs of Swi6 and Rlm1 have been functionally analysed (Ding et al., 2018, 2020). Swi6 is crucial, but Rlm1 is dispensable for vegetative growth of Foc; however, deletion of *SWI6* or *RLM1* results in both reduced virulence in banana plantlets and increased sensitivity to hydrogen peroxide. Interestingly, both Swi6 and Rlm1 are involved in regulation of fusaric acid biosynthesis genes, and Rlm1 is also required for transcription of beauvericin biosynthesis genes, suggesting that Swi6 and Rlm1 may also take part in regulation of secondary metabolism in Foc. In *F. graminearum*, FgRlm1 and FgSwi6 are required for fungal growth, development, pathogenicity, and secondary metabolism processes, suggesting a conserved role of Rlm1 and Swi6 homologs among *Fusarium* fungi (Liu et al., 2013; Yun et al., 2014).

The Fmk1 MAPK pathway is highly conserved in fungi and is essential for infection in most plant pathogens (Turra et al., 2014). In Fol, the Fmk1 MAPK cascade promotes expression of the TF Ste12 to activate transcription of genes involved in pathogenicity (Rispail & Di Pietro, 2009). Targeted deletion mutants lacking Ste12 showed dramatic defects in invasive growth, vegetative hyphal fusion, and secretion of pectinolytic enzymes as well as reduced virulence in tomato plants (Rispail & Di Pietro, 2009). Additionally, it has been shown that Ste12 positively regulates extracellular amylase and cellulase activities but is not impaired in the activation of pectinase genes in Fol (Rispail & Di Pietro, 2009). To date, except for Ste12, the other downstream TFs of Fmk1 signalling pathways have remained unidentified in *F. oxysporum*. In *S. cerevisiae* and other filamentous fungi, Ste12 is required for mating. Loss of Ste12 leads to defects in the sexual cycle of the plant pathogens *Cryphonectria parasitica* (Deng et al., 2007) and *B. cinerea* (Schamber et al., 2010), the human pathogens *C. albicans* (Liu et al., 1994) and *C. neoformans* (Yue et al., 1999), and the saprophytes *N. crassa* (Li et al., 2005) and *A. nidulans* (Vallim et al., 2000). The sexual spores (ascospores) produced by some *Fusarium* species function as infectious propagules; however, sexual reproduction in *F. oxysporum* is still unknown.

## 5 TRANSCRIPTIONAL REGULATION OF ASEXUAL CONIDIATION IN *F. OXYSPORUM*

*F. oxysporum* is unique in its asexual reproduction, producing large quantities of three types of conidia (microconidia, macroconidia, and chlamydospores) (Ma et al., 2013). Observation of host colonization suggests that this process is expedited by the accumulation of conidia, which move freely within the xylem, leading to blockade of the vessels by forming a plate at the end. Then, microconidia will germinate and form germ tubes that are able to produce more conidia (Beckman et al., 1961). The germination, penetration, and sporulation series accelerates the speed of host colonization. StuA and Ren1 regulate the development of asexual conidiation in *F. oxysporum*. In *F. oxysporum* f. sp. *melonis* (Fom) *REN1* deletion mutants, the production of microconidia and macroconidia are affected, but normal vegetative growth and chlamydospore formation are observed. In Fol, deletion of *STUA* showed normal microconidium formation and reduced macroconidium formation but increased chlamydospore formation under culture conditions, but the mutants produced markedly fewer macroconidia and microconidia in infected plants than the wild‐type strain. However, loss of Ren1 or StuA did not affect pathogenesis, suggesting that microconidia and macroconidia may not be required for *F. oxysporum* pathogenicity.

## 6 TRANSCRIPTIONAL REGULATION OF GENERAL METABOLISM AND VIRULENCE IN *F. OXYSPORUM*

Some TFs are specific for virulence, but some TFs affect many developmental processes, acting as general regulators in fungal biological processes. The three TFs, Ebr1, Con7‐1, and Snt2, affect general metabolism, which leads to weak fitness of fungi. The basic phenotypes, including virulence, were affected after loss of these three factors. However, whether these three TFs also have direct roles in virulence regulation is still unknown. In contrast to these three TFs, Fow2 and Cti6 are specific regulators of the pathogenicity of *F. oxysporum*, and their loss affects virulence, but the mechanism is also unknown.

Ebr1 (enhanced branching 1) has been found to impair growth, reduce pathogenicity, and slightly reduce biocontrol capacities in *F. graminearum* (Thatcher et al., 2012; Zhao et al., 2011). Interestingly, *EBR1* is present as multiple copies in Fol strains, while only a single copy is found in *F. graminearum* (Thatcher et al., 2012; Zhao et al., 2011). In the Fol 4287 strain, nine paralogs of Ebr1 were retrieved, among which Ebr1 (FOXG_05408) showed the highest similarity to FgEbr1 (89%). Deletion of *EBR1* in different Fol strains containing different numbers of *EBR* genes resulted in similar impaired growth and reduced pathogenicity, suggesting that loss of *EBR1* causes a growth and pathogenicity phenotype in Fol strains independent of the other *EBR* copies (Thatcher et al., 2012).

Con7 has been described as a central regulator of infection‐related morphogenesis in the rice blast fungus *M. oryzae* (Odenbach et al., 2007). However, three copies of Con7 homologs have been identified in Fol, and Con7‐1 (FOXG_11503) shares the closest (69%) identity with Con7 of *M. oryzae* (Ruiz‐Roldan et al., 2015). Gene deletion and comparative microarray‐based gene expression analysis demonstrated that Con7‐1 acts as a general regulator in polar growth, hyphal branching, conidiation, and pathogenicity (Ruiz‐Roldan et al., 2015). In contrast, simultaneous inactivation of both Con7‐2 copies caused no detectable defects in the deletion mutants, suggesting that the other two Con7‐2 copies are not important for the growth, development, and pathogenicity of *F. oxysporum*.

Snt2 is involved in regulation of hyphal growth, hyphal septation, conidiation, and fungal respiration in *F. oxysporum* f. sp. *melonis* (Fom) (Denisov et al., 2011). *SNT2* deletion mutants showed obviously reduced virulence in muskmelon plants and exhibited significantly lower colonization of upper plant stems, suggesting that Snt2 is required for the early stage and host colonization of Fom pathogenesis (Denisov et al., 2011). Furthermore, Snt2 has been found to regulate the expression of four genes (*IDI4*, *PDC*, *MSF1*, *eEF1G*) in the TOR pathway, but Snt2 is not involved in nitrogen metabolism, suggesting that the Snt2 and TOR pathways share common components to support an overlapping regulatory mechanism in maintaining cell biological processes in *F. oxysporum* (Denisov et al., 2011).

Fow2, a Zn(II)2Cys6‐type TF, is required for full virulence but not hyphal growth and conidiation in Fom (Imazaki et al., 2007). Fow2 also controls the ability of Fom to invade roots and colonize plant tissue (Imazaki et al., 2007). *FOW2* is highly conserved in formae speciales of *F. oxysporum*. Homologs of Fow2 were identified in six other formae speciales of *F. oxysporum*, and the deletion of *FOW2* in Fol also resulted in near nonvirulence in tomato plants (Imazaki et al., 2007). Cti6, a PHD finger‐containing TF, is also required for full pathogenicity of Fol toward tomato plants and participates in chromatin modifications (Michielse, van Wijk, Reijnen, Cornelissen, et al., 2009). In *S. cerevisiae*, Cti6 participates in securing rapid GAL1 transcriptional activation by acting in concert with the histone acetyltransferase‐containing complex SAGA to alleviate Cyc8‐Tup1‐mediated repression (Han & Emr, 2011). To date, the downstream targets of Cti6 and Fow2 remain unknown in *F. oxysporum*, thus meriting further analysis.

## 7 CONCLUSION

**FIGURE 1:**
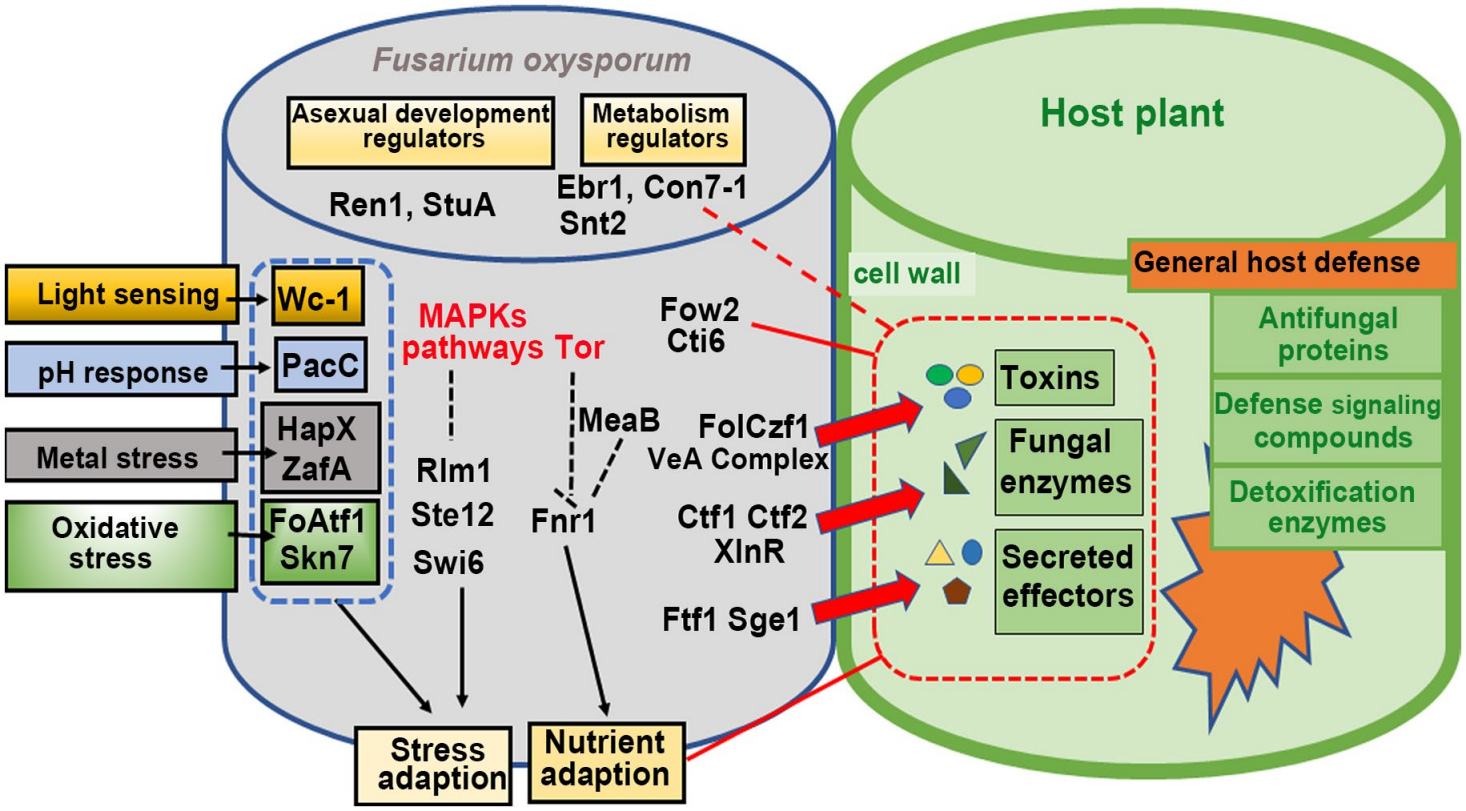
Schematic representation of the potential relationship of the characterized transcription factors with the different signalling pathways and cellular processes associated with *Fusarium oxysporum* virulence

The lack of effective control methods for Fusarium wilt disease threatens the production of many economic crops, especially for cultivation in a greenhouse. Thus, an improved understanding of the molecular mechanisms underlying the host pathogenicity of *F. oxysporum* is critical for developing sustainable control methods for this disease. *F. oxysporum* enters the plants directly through roots via infectious hyphae and then colonizes and proliferates in xylem vessels (Yadeta & Thomma, 2013). Directed virulence factors, including CWDEs, mycotoxins, and effectors, have been demonstrated to be important for the *F. oxysporum* infection process and here we have summarized the TFs that are responsible for regulation of these virulence factors in *F. oxysporum* (Figure 1 and Table 2). Clearly, the genes encoding important virulence factors are regulated by more than one TF, for example the TFs Ftf1 and Sge1 are both involved in regulation of Six family genes (van der Does et al., 2016; Michielse, van Wijk, Reijnen, Manders, et al., 2009; Ramos et al.,^2007^; de Vega‐Bartol et al., 2011). Six effectors are specifically expressed in the infection process, but how Ftf1 and Sge1 cooperate and promote the expression of *SIX* genes in the infection process is still unknown; thus, genome epigenetic analysis and detailed functional mechanism analysis for these important virulence‐related TFs are needed.

During the conidial germination and penetration stage, *F. oxysporum* is considered to be nutrient starved and invades plant hosts to obtain the required nutrient supplies (López‐Berges, 2020; López‐Berges et al., 2010, 2012). For survival and proliferation in xylem vessels, *F. oxysporum* effectively utilizes the available nutrition in the xylem and responds to stresses from surroundings, such as light sensing, pH response, iron starvation, zinc homeostasis, and oxidative stress, which play an important role in the infection process. As shown in Figure 1, the known TFs involved in regulation of the above process are summarized in this review. However, our understanding of this process is still limited. To further understand the stress‐responsive gene regulatory networks in *F. oxysporum*, components of the regulatory systems should be identified, including genes encoding TFs and genes encoding downstream component products.

The availability of the complete genomes of some formae speciales of *F. oxysporum* may help in improving understanding of the TFs involved in pathogenesis and has already provided evidence of several potential pathogenic mechanisms (Guo et al., 2014; Ma et al., 2010). Some of these TFs have been predicted to have essential roles in coordinating gene expression and might coordinate the exchange between core chromosomes and accessory chromosomes. Among the 26 functionally analysed TFs in *F. oxysporum*, *FTF1*, *PACC*, *EBR1*, and *FOW2* are expanded in the genome and contribute to pathogenicity (Caracuel et al., 2003; Jonkers et al., 2014; Ramos et al., 2007). Interestingly, *FTF1* has the largest number of copies (11 genes predicted in Fol and four genes predicted in Fop), while *EBR1* and FOW2 have nine and three gene copies in the Fol genome, respectively (Jonkers et al., 2014; Ramos et al., 2007). A recent study investigating the genome of the opportunistic fungal pathogen *F. oxysporum* NRRL 32931 identified four paralogs of *PACC* (*PACC_0*, *PACC_a*, *PACC_b*, and *PACC_c*) (Zhang et al., 2020). These four TFs comprise a single‐copy gene found on core chromosomes, with several copies distributed on accessory chromosomes. TFs encoded in the core genome have been demonstrated to potentially regulate the expression of other accessory chromosome paralogs, such as *EBR1*‐like paralogs (*EBR2*, *EBR3*, and *EBR4*), which are regulated by core chromosome *EBR1* (Jonkers et al., 2014). Similar to *EBR*, core chromosome *PACC* plays a predominant function and has been found to regulate alkaline pH (Zhang et al., 2020). In contrast, the deletion accessory chromosome *FTF1* copy displayed a role in virulence, while core chromosome *FTF1*, up‐regulated during infection, showed less effect on virulence (de Vega‐Bartol et al., 2011; Niño‐Sánchez et al., 2015). The study also revealed that *FTF1* on either accessory chromosomes or the core chromosome can be induced to regulate many effectors in Fol, suggesting that accessory chromosome and core chromosome TFs may play various functions (van der Does et al., 2016). Although paralogous TFs share the same DNA‐binding motifs, they perform distinct regulatory functions. Further genomic analysis and functional studies of TF expansion can give rise to new TF families and their function in the origin and evolution of pathogenesis.

In this review, we have summarized the pathogenicity‐related TFs that have been characterized in *F. oxysporum* (Figure 1 and Table 2). However, our understanding of the role of TFs that modulate *F. oxysporum* virulence is far from complete, and particularly lacking are those involved in the early stages of host–pathogen interactions and the regulatory network of virulence‐related factors, thus further exploration of the mechanism and target networks of the known TFs is still needed.

## Data Availability

Data sharing is not applicable to this article as no new data were created or analysed in this study.
